# WhatsApp in hospital? An empirical investigation of individual and organizational determinants to use

**DOI:** 10.1371/journal.pone.0209873

**Published:** 2019-01-11

**Authors:** Anna De Benedictis, Emanuele Lettieri, Cristina Masella, Luca Gastaldi, Giordana Macchini, Camilla Santu, Daniela Tartaglini

**Affiliations:** 1 Department of Healthcare Professions, Hospital General Management, University Hospital Campus Bio-Medico, Rome, Italy; 2 Faculty of Medicine & Surgery, University Campus Bio-Medico, Rome, Italy; 3 Department of Economics, Management and Industrial Engineering, Politecnico of Milan, Milan, Italy; National Institute of Health, ITALY

## Abstract

The increasing use of messaging applications such as WhatsApp for both social and personal purposes has determined an increase in the widespread use of these technologies, even in healthcare. A growing number of healthcare professionals have adopted WhatsApp in their daily work in order to share information with peers and patients. Past research has highlighted the advantages and disadvantages of WhatsApp usage in healthcare settings; in particular two positions appear to coexist in the scientific debate: those that expose and underline all of the positive aspects of the phenomenon, and those which also highlight the negative aspects, linked in particular to the clinical risks for patients, data security and privacy protection. The main objective of this study was to assess if and how individual and organizational determinants can trigger or inhibit the use of WhatsApp in a hospital setting, and which variables managers can exploit to guide professionals’ behaviors. Data were collected through a survey administered to physicians and nurses in an Italian University Hospital in Rome; a total of 191 high-quality responses were received. The results show that WhatsApp is widely used in the Hospital, and that its use is mainly due to the perception of numerous advantages and benefits reported in clinical practice. Moreover, an interplay exists between organizational and individual factors in determining the use of WhatsApp between healthcare professionals and with patients. In particular, individual factors play a key role as determinants of the use of WhatsApp; healthcare professionals use this technology mainly based on its perceived usefulness. Instead, organizational factors play a secondary role; they do not have a direct influence on the use of WhatsApp, but always act through individual factors. This study is the first to analyses the influence of individual and organizational determinants of WhatsApp usage in the hospital setting, and provides hospital managers with important information in order to manage this phenomenon and implement adequate strategies to exploit its potential increase.

## Introduction

The increasing use of messaging applications such as WhatsApp for both social and personal purposes has determined an increase in the widespread use of these technologies in healthcare [1–6]. A growing number of healthcare professionals have adopted WhatsApp in their daily work in order to share information with peers and patients [7–11]. Past research has highlighted the advantages and disadvantages of WhatsApp usage in healthcare. In this regard, two positions appear to coexist in the scientific debate: those that expose and underline all of the positive aspects of the phenomenon [6–16], and those which highlight the negative aspects, linked in particular to the clinical risks for patients, data security and privacy protection [8,16–21].

Some of the main advantages of using WhatsApp in healthcare are as follows: improvement of communication [22]; no requirement for a computer [23,24]; time saving [6,15]; possibility of an immediate response [20,25]; improvement of surgery performance and reduction of consultation time [25,26]; smoothing of hierarchy [8]; and the encouragement of junior doctors to seek help and improve the team perception of effectiveness [19]. On the other hand, existing risks or disadvantages have also been reported: increase in workload, disparity in the sense of urgency, worsening of professional relationships and risk of unprofessional behavior [18]; need to stay online 24 hours a day; impossibility to print a record of the chat; clinical information not being included in medical records; difficulty identifying patients in chats [16,19]; possible issues of privacy and data protection [20]; and the risk of reducing the autonomy of junior doctors [8]. Despite the many benefits, WhatsApp is used by professionals without political strategies, so it is necessary to develop guidelines for its usage by interdisciplinary groups and for communication between patients and professionals [27]. Hospitals are increasingly looking to evaluate the impact of WhatsApp usage on care delivery [11]; however, there is still very limited evidence regarding if and how individual and organizational determinants can trigger or inhibit such phenomenon.

The main objective of the study is to assess if and how individual and organizational determinants can trigger or inhibit the use of WhatsApp in a hospital setting, and which variables managers can exploit to guide professionals’ behaviors. In particular, the following research questions have been investigated:

- In what way is WhatsApp used in hospital settings by physicians and nurses with patients and between colleagues?
- Which are the main perceived benefits and threats concerning the use of WhatsApp in a hospital setting by physicians and nurses?
- Which are the determinants (individual and/or organizational) of the use of WhatsApp in a hospital setting?
- Is there an interplay between individual and organizational determinants?

### Theoretical background

**Fig 1:**
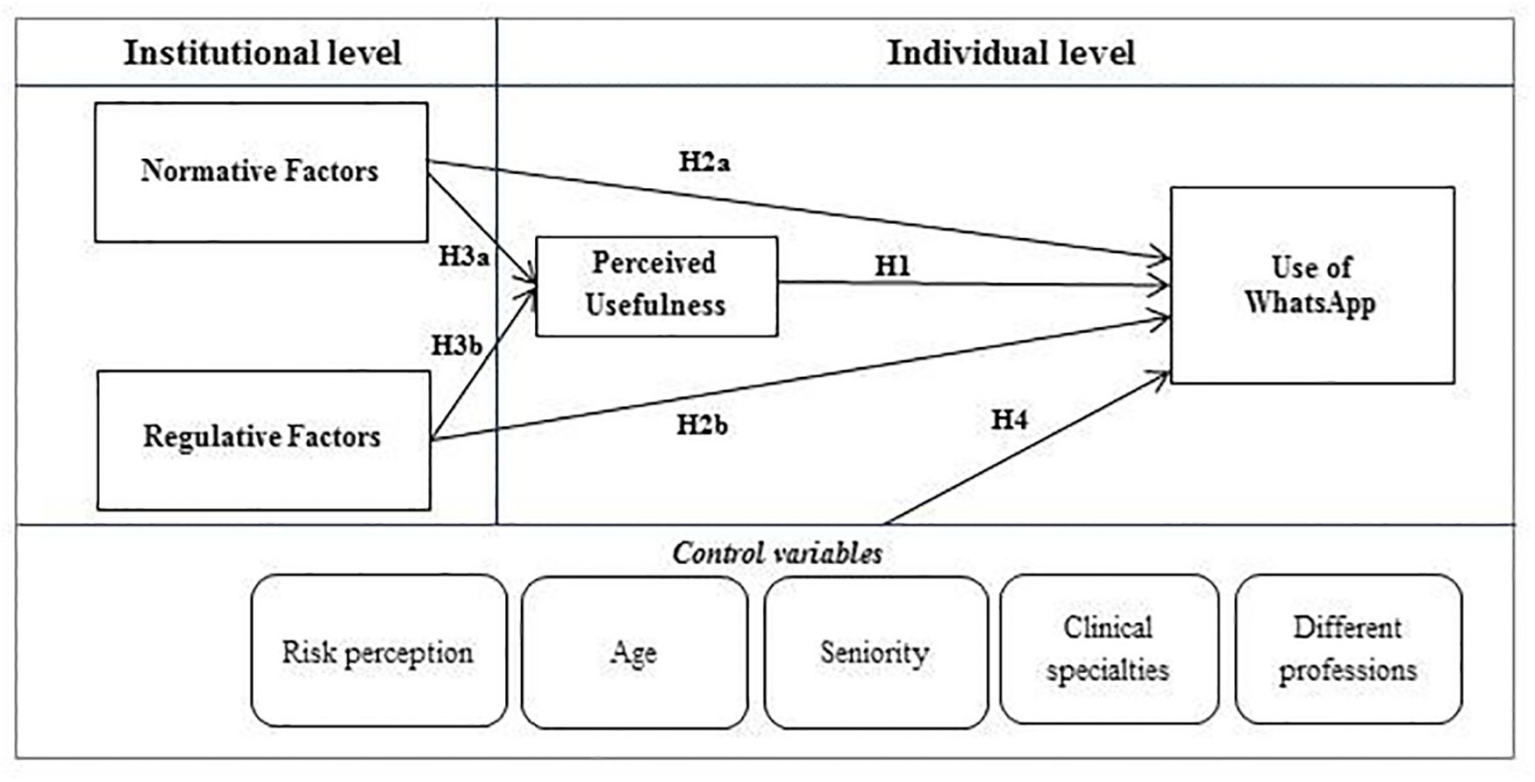
Theoretical framework.

In order to evaluate the interplay between individual and organizational variables, it was necessary to create a theoretical model that could explain this phenomenon (Fig 1). In particular, we drew inspiration from two well-established and respected theories:

- *Technology Acceptance Model* (TAM): that has been widely used in the last decades in healthcare in order to understand what leads people to accept or reject information technology [28, 29];
- *Institutional Theory*, which explains how “institutional”–in our case, “organizational”–forces shape organizations and professionals’ behaviors [30–32].

These theories are introduced briefly in the followings.

#### Technology Acceptance model

The TAM theory was introduced for the first time by Davis in 1989 [28]. The main problem raised by the author was understanding what leads people to accept or reject information technology. In this regard, two explanatory factors have been identified: the perceived usefulness and the perceived ease of use. Perceived usefulness measures “the degree to which a person believes that using a particular system would enhance his or her job performance” [28], and therefore induces individuals to use technology as it allows them to obtain better results. On the other hand, the perceived ease of use measures “the degree to which a person believes that using a system would be free of effort” [28, 29] and induces the potential users to adopt a certain technology since it requires low energy expenditure. TAM adopted these explanatory factors from other previous theories, in particular, the Theory of Reasoned Action (TRA) and the Theory of Planned Behavior (TPB). In the last years, TAM has undergone a number of modifications that resulted in different models, such as TAM2, which adds a variable about the social influence towards adoption, and UTAUT, which reasons about the influence of performance expectancy. For the sake of our study, we relied upon the original model, which is still the most commonly used and consistently proved as effective. Additionally, the potential role of social influence has been captured by the inclusion in our model of organizational factors.

In this study we decided to include in our explanatory framework only the variable “perceived usefulness” because from preliminary interviews and past experiences we know that all physicians and nurses included in this Hospital use smartphones and WhatsApp daily, so we excluded any problems related to the digital divide. Moreover, such digital literacy in the use of WhatsApp has been confirmed by the study.

#### Institutional Theory

Institutional Theory refers to a stream of organizational research that recognizes the significant organizational effects that are associated with an increase in cultural and social forces. According to Scott [30–32], “Institutions are made up of cultural-cognitive, normative and regulative elements, which together with associated activities and resources offer stability and meaning to social life.” These three forces are present in totally developed institutional systems, with economists and political scientists placing emphasis on regulative, sociological and normative factors, and anthropologists and organizational theorists placing emphasis on cognitive-cultural factors. According to this perspective, individuals are embedded in institutional pillars that limit the scope of their rational assessment, and direct the engagement of specific behaviors [30–32]. Scott [30–32] defines the three “institutional pillars” as follows:

- *Regulative pillar*: which regards the existence of regulations, rules and processes whose breach is monitored and sanctioned;
- *Normative pillar*: which introduces a social dimension of appropriate behaviors in the organization;
- *Cultural pillar*: which emphasizes the use of common schemas, frames, and other shared symbolic representations that create an attachment to the ‘appropriate’ behavior.

In this study we decided to include in our explanatory framework only the regulative and normative pillars, since, being a single center study, we were not able to appreciate significant differences in the cultural pillar. Further multicenter studies should add this additional organizational explanatory variable.

### Theoretical framework

Consistent with the research questions and taking inspiration from the theories described above, a theoretical framework has been defined, in which it is assumed that individual and organizational determinants are combined together to explain the use of WhatsApp between healthcare professionals and with patients in a hospital setting. Coherently with past research on user acceptance models [33,34], we added some control variables that are considered able to affect the results; they are: risk perception, age, seniority, clinical specialties and profession. (Fig 1)

According to the research questions and the theoretical model the following research hypotheses (H) were stated: H1: Perceived usefulness directly affect the use of WhatsApp; H2a: Normative factors directly affect the use of WhatsApp; H2b: Regulative factors directly affect the use of WhatsApp; H3a: Normative factors directly affect the perceived usefulness of WhatsApp; H3b: Regulative factors directly affect the perceived usefulness of WhatsApp; H4: Control variables (risk perception, age, seniority, clinical specialties and different professions) affect the use of WhatsApp. Hypotheses 3a and 3b are the most relevant to the study, since they explore if and how the individual and organizational variables interact and which of these variables are dominant. All hypotheses regarding the use of WhatsApp with patients and colleagues were tested to assess whether or not the interplay between variables is the same.

## Materials and methods

### Setting and research methodology

A survey was designed and administered in an Italian University Hospital, in Rome. The unit of analysis is the group of healthcare professionals (both nurses and physicians) of the Hospital. The questionnaire (S1 Table) was designed based on the scales identified in the literature and reviewed in detail by the group of researchers. Moreover, a pilot test of the questionnaire was carried out before the survey. The questionnaire consists of three main sections: the use of WhatsApp; scales and constructs of the proposed model; control variables and characteristics of the respondents. The use of WhatsApp was evaluated through the following macro-constructs, including 30 items: personal use of WhatsApp in daily life, use of WhatsApp with patients, and use of WhatsApp with other healthcare professionals [1,35]. Individual variables were evaluated by 15 items, in particular, the scale for the measurement of perceived usefulness was adapted from the studies by Venkatesh [36–39]. Organizational variables were explored through 11 items related to regulative and normative factors. The scale for the measurement of regulative and normative factors has been adapted from the study by Scott [40]. The survey items are listed in the questionnaire (S1 Table). Moreover, the risk perception related to the use of WhatsApp in a hospital setting was explored by 12 items. Additional questions have been designed to gather demographic and sample information. All of the questionnaire items were explored using a 7-point Likert scale, with 1 indicating “totally disagree” and 7 “totally agree”, or a 5-point Likert-like scale with 1 indicating “never” and 5 “always”. The completion rate was assessed weekly. The first re-call was made one week after the expiration date for compilation. Two or three days after the first follow-up, the second recall was sent, and two or three days after the second follow-up, the third recall was sent.

### Statistical analysis

Statistical analysis was performed using the software Stata 14.1. The internal consistency of the constructs was verified through the Cronbach’s Alpha. The correlation between professional role (doctors vs. nurses) as well as the answers provided for each item were analyzed through the Fisher’s test. A p-value of <0.05 was considered significant. Moreover, a path analysis was performed in order to test the proposed model.

## Results

All questionnaires were completed in the period between February and September 2017, and a total of 191 responses (125 nurses and 66 physicians) were received (30.3%). Three follow-ups were sent to nurses and three to physicians (Table 1). The characteristics of respondents are described in Table 1.

**Table 1:** Characteristics of respondents.

		Frequency	Percentage
Gender	Male	63	33
	Female	128	67
Age	21–30	45	23.6
	31–40	81	42.4
	41–50	43	22.5
	> 50	22	11.5
Profession	Physician	66	34.6
	Nurse	125	65.4
Seniority (years of working experience)	0–10	101	52.9
	11–20	59	30.9
	21–30	19	9.9
	31–40	10	5.2
	> 40	2	1.0

### WhatsApp usage

Data confirm the widespread use of WhatsApp by the doctors and nurses included in the study, both in their personal life and in the workplace. WhatsApp usage in personal life is very common; nurses and physicians use it in order to participate in group discussion, send private messages to other people, send written messages or send images. Instead, the use of WhatsApp to organise agendas with others, to send audio notes or to share moments of life with others is less frequent (S2 Table). In the hospital setting, WhatsApp is used for different reasons, between colleagues and with patients. Data show that a statistical correlation exists between the use of WhatsApp in the clinical setting and the profession. In particular, physicians, more than nurses, use WhatsApp to share scientific information (p = 0.038), manage and share agendas (p = 0.001), communicate about clinical situations (p<0.0001), ask for information or give directions (p = 0.042), send patient data in the form of images or videos (p = 0.042), receive patient information from other hospitals (p = 0.001) (S3 Table). Some physicians report that patients often ask them to use WhatsApp to facilitate communication, and send images or videos to get an evaluation before a visit, or without having a scheduled visit. Nurses’ behaviors are very different: almost none of the interviewed nurses use WhatsApp to communicate with patients (p<0.0001), only a few nurses report that patients ask them to use this App to facilitate communication (p<0.0001), and the number of nurses who suggest using WhatsApp to patients is less than 5% (p<0.0001) (S4 Table). Respect to nurses, physicians use WhatsApp more frequently in order to: organize the agenda with patients (p<0.0001); send to patients results of diagnostic tests (p = 0.001); monitor chronic patients’ clinical conditions (p<0.0001); to answer urgent questions of patients (p<0.0001). Many perceived benefits are reported; in fact, data suggest that the use of WhatsApp: improve communication between professionals and doctor-patient relationship; increase efficiency; can reduce the costs in the Hospital; is time saving; improve the sharing of clinical and scientific knowledge; might improve performances of research and teaching activities. At the same time, some respondents suggest that the use of WhatsApp at work can reduce productivity (e.g. *I am distracted by other factors that do not concern my job*), or can increase the workload (S5 and S6 Tables), and that it could generate several risks for both healthcare professionals and patients. Respect to nurses, physicians report more frequently that WhatsApp usage in the clinical setting is risky because no guidelines or recommendations are available (p = 0.005), and because it can compromise the patient-physician relationship (p<0.0001). Moreover, professionals agree with the assumption that the transmission of sensitive patients’ data through WhatsApp should provide the patient’s informed consent for data treatment (S7 and S8 Tables).

### Testing the theoretical framework

#### Questionnaire’s constructs internal consistency

The internal consistency of the questionnaire’s constructs was verified through the Cronbach’s Alpha (Table 2). Values greater than or equal to 0.7 were considered acceptable.

**Table 2:** Questionnaire’s constructs validity analysis.

Variable/Macro-item	Cronbach’s Alpha
Use	0,92
Perceived Usefulness	0,81
Normative Factors	0,79
Regulative Factors	0,77
Perceived Risk	0,93

#### Determinants of current behaviors

**Fig 2:**
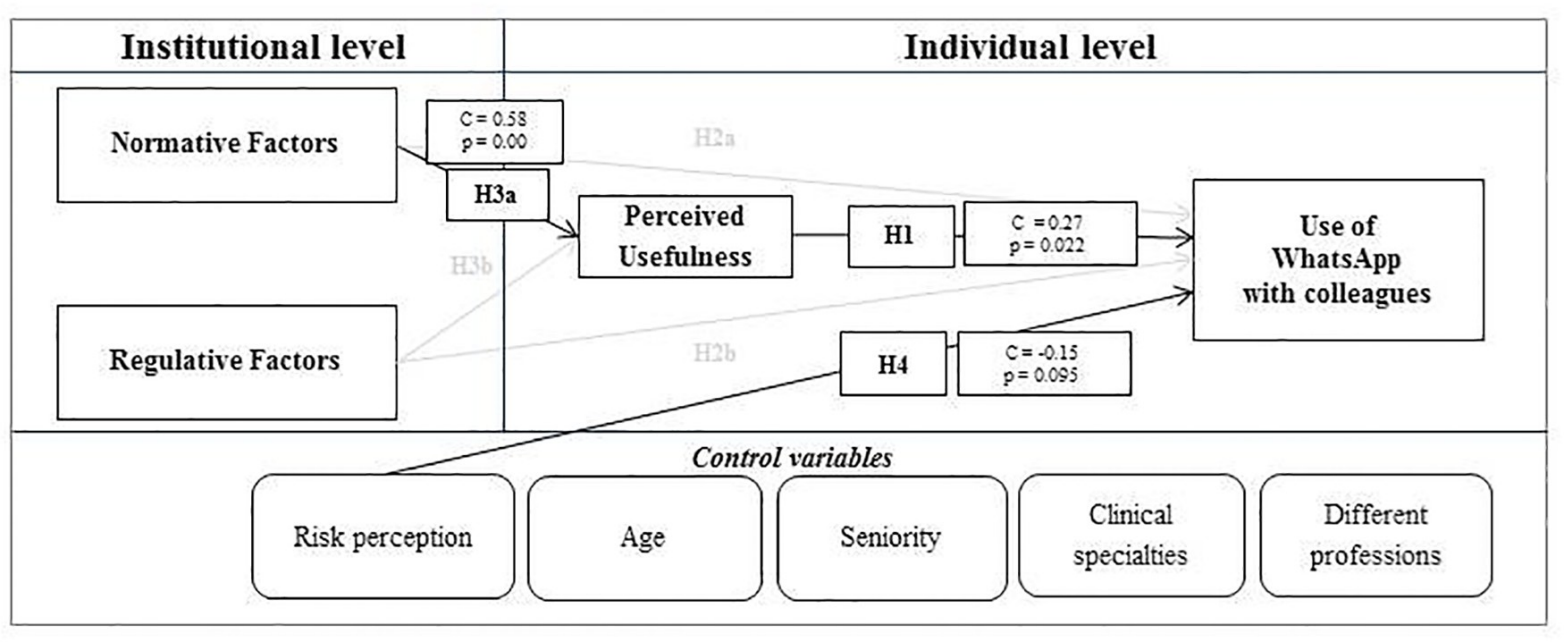
Determinants of current behaviors between professionals.

**Fig 3:**
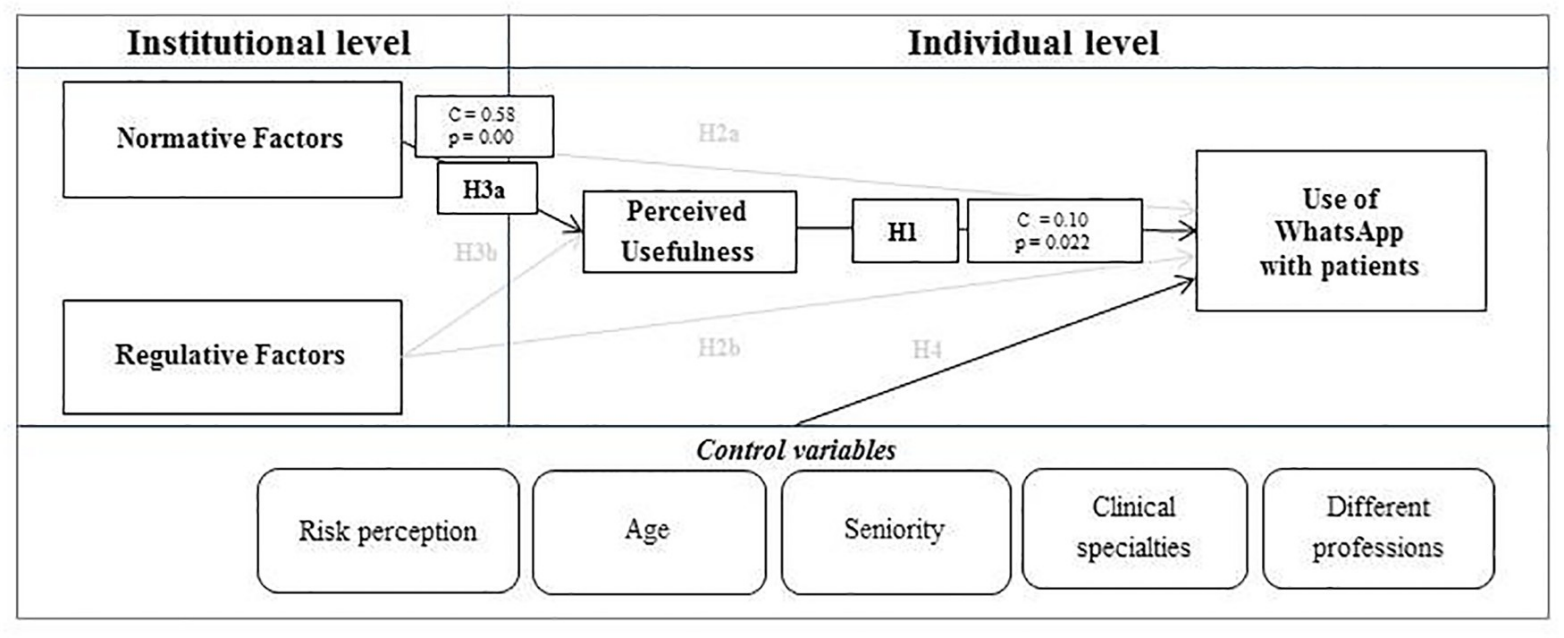
Determinants of current behaviors with patients.

Thanks to a Path Analysis performed within the SEM builder environment, it was possible to verify the proposed model and evaluate the main determinants (individual and organizational) of the use of WhatsApp by the doctors and nurses included in the study. Results suggest that regulative factors do not have an impact on the use of WhatsApp with patients or colleagues, while normative factors have a direct impact on the perceived usefulness of WhatsApp. Moreover, perceived usefulness is directly related to the use of WhatsApp with both patients and colleagues, and risk perception is negatively related to the use of WhatsApp with colleagues. (Table 3, Figs 2 and 3)

**Table 3:** Determinants of current behaviors.

Research Hypotheses	Use of WhatsApp between professionals	Use of WhatsApp with patients
RH1: *Perceived Usefulness* directly affect the *use of WhatsApp*.	Coeff = 0.27 p** = 0.022	Coeff = 0.10 p** = 0.022
RH2a: *Normative factors* directly affect the *use of WhatsApp*.	p = 0.723 NS	p = 0.25 NS
RH2b: *Regulative factors* directly affect the *use of WhatsApp*.	p = 0.436 NS	p = 0.582 NS
RH3a: *Normative factors* directly affect the *perceived usefulness* of WhatsApp.	Coeff = 0.58 p*** = 0.00	Coeff = 0.58 p*** = 0.00
RH3b: *Regulative factors* directly affect the *perceived usefulness* of WhatsApp.	p = 0.70 NS	p = 0.68 NS
RH4: *Risk perception* affect the *use of WhatsApp*.	p* = 0.095 Coeff. = -0.15	p = 0.884 NS
RH4: Other control variables (*Age*, *seniority*, *clinical specialties and different professions)* affect the *use of WhatsApp*.	NS	NS

NS = Not Significant,

*p value<0.1,

**p value<0.05,

***p value<0.005

The main results of the impact of individual and organizational determinants on WhatsApp usage between colleagues and with patients are reported in Figs 2 and 3. In both cases, normative factors (e.g. colleagues’ behaviors and patients’ preference) directly influence the perceived usefulness of WhatsApp, while individual factors (perceived usefulness) directly influence WhatsApp usage.

## Discussion

This study is the first to analyse the influence of individual and organizational determinants on the use of WhatsApp in a hospital setting. The findings confirms that WhatsApp is increasingly used in personal life and in the hospital environment by doctors and nurses in order to communicate and share data between peers and patients [11,15]. Also, its usage is mainly due to the perception of numerous advantages and benefits reported in clinical practice [6–16] and particularly related to the perception of greater ease in communication and to a leaner management of some processes. However, healthcare professionals’ behaviors do not appear to be uniform. In fact, compared to doctors, nurses rarely use WhatsApp in order to communicate with patients or share clinical information between colleagues. On the other hand, the use of WhatsApp is perceived to not be safe for both patients and professionals [8 16–21], and its usage is inversely related to the perceived risk. At the same time, while nurses and physicians consider WhatsApp not safe, they use it anyway in their clinical practice with both, colleagues and patients. For this reason, we assume that the use of WhatsApp in a healthcare setting can be considered an extreme case of “back-door adoption”, which is the case for technologies that are so easy to use that they are diffused without discussion or a prior policy definition, and which are brought by healthcare professionals without any formal approval or assessment from top managers about the opportunities and risks that these innovations might bring along with them [41]. Furthermore, this phenomenon could be particularly dangerous because of its speed and uncontrolled spread in a very peculiar and complex context, where even small process variables can negatively and harmfully affect clinical risk for patients.

The findings show an interplay between organizational and individual factors in determining the use of WhatsApp in the healthcare context between healthcare professionals and with patients. In particular, individual factors play a key role as determinants of the use of WhatsApp; healthcare professionals use this technology mainly based on its perceived usefulness. Instead, organizational factors play a secondary role; they do not have a direct influence on the use of WhatsApp, but always act through individual factors. Between organizational factors, the regulative ones (e.g. rules imposed by management) have no influence on the use of WhatsApp, while normative ones (e.g. the influence of colleagues or patients) have a positive impact. From these first results emerges the importance of managers, researchers and policy makers working to regulate a phenomenon that, while it is considered useful and effective, is widespread without shared rules. The fact that the regulatory factors have no impact on the use of WhatsApp is probably related to the lack of clear rules for professionals. Management interventions must therefore be aimed at the regulation of its use, and focused on training and information for doctors, nurses and patients with respect to the risks and benefits of this tool, rather than the complete prohibition of the same, a strategy that would be useless and counterproductive.

The results of the study are valid, with several limitations. First, it was a single center survey conducted with a relatively small number of healthcare professionals, although they were representative of all Hospital departments. The number of nurses who answered the questionnaire was higher than the number of physicians, even though, despite the relatively small response rate, the number of respondents was high. This was probably due to the size of the Hospital and the accessibility to personal email of healthcare professionals.

## Conclusion

This study furthers current knowledge about digital innovation implementation in a professional setting with a focus on “back door” adoption. In particular, by combining organizational and individual factors in a coherent theoretical framework, the study showed connections of different factors as well as their independent effect on the adoption of “employee driven” innovation, and shed new light on factors that can help managers to oversee this phenomenon and implement adequate strategies to exploit its potential increase at the same time as the level of safety for the patients. It would be desirable to continue the study by involving a larger number of hospitals to test the model and make the data more generalisable. From this study, some possible future steps for practitioners and researchers in this area also emerge. First, it is important to define some guidelines for WhatsApp usage in the healthcare setting. Another important point would be verifying the level of evidence of recommendation for the use of WhatsApp with patients in specific clinical settings and the outcomes on patient care and Key Performance Indicators that are directly related to the use of WhatsApp. In fact, despite the numerous perceived benefits, only a few studies are available about the level of evidence of recommendations regarding the use of WhatsApp in clinical and care settings. This aspect is particularly interesting especially in the medical setting, showing why it is so important to work based on scientific evidence and recommendations.

## Supporting information

S1 Table Questionnaire. (DOCX)

S2 Table Personal use of WhatsApp. (DOCX)

S3 Table Mode of WhatsApp usage between healthcare professionals. (DOCX)

S4 Table Mode of WhatsApp usage with patients. (DOCX)

S5 Table Perceived benefits about WhatsApp usage between healthcare professionals. (DOCX)

S6 Table Perceived benefits related to WhatsApp usage with patients. (DOCX)

S7 Table Perceived threats about WhatsApp usage between healthcare professionals. (DOCX)

S8 Table Perceived threats related to WhatsApp usage with patients. (DOCX)

## References

[1] Mobasheri MH, King D, Johnston M, Gautama S, Purkayastha S, et al The ownership and clinical use of smartphones by doctors and nurses in the UK: a multicentre survey study. BMJ Innovation. 2015;00:1–8.

[2] Wolf JA, Moreau JF, Akilov O, Patton T, English JC, Ferris LK, Diagnostic inaccuracy of smartphone applications for melanoma detection. JAMA Dermatology. 2013; 149:422–6. 10.1001/jamadermatol.2013.2382 23325302 PMC4019431

[3] Haffey F, Brady RR, Maxwell S. A comparison of the reliability of smartphone apps for opioid conversion. Drug Safety. 2013;36:111–17. 10.1007/s40264-013-0015-0 23322549

[4] Martinez-Perez B, De La Torre-Diez I, Lopez-Coronado M. Privacy and security in mobile health apps: a review and recommendations. Journal of Medical Systems. 2015;39:181 10.1007/s10916-014-0181-3 25486895

[5] Dorwal P, Sachdev R, Gautam D, Jain D, Sharma P, Tiwari AK, et al Role of WhatsApp Messenger in the Laboratory Management System: A Boon to Communication. Journal of Medical Systems. 2016;40(1):14 10.1007/s10916-015-0384-2 26573651

[6] Gulacti U, Hatipoglu S, Polat H. An Analysis of WhatsApp Usage for Communication Between Consulting and Emergency Physicians. Journal of Medical Systems. 2016;40(6):130 10.1007/s10916-016-0483-8 27083574

[7] Johnston MJ, King D, Arora S, Behar N, Athanasiou T, Sevdalis N, Darzi A, et al Smartphones let surgeons know WhatsApp: An analysis of communication in emergency surgical teams. Am. J. Surg. 2015;209(1):45–51. 10.1016/j.amjsurg.2014.08.030 25454952

[8] Wani SA, Rabah SM, AlFadil S, Dewanjee N, and Najmi Y. Efficacy of communication amongst staff members at plastic and reconstructive surgery section using smartphone and mobile WhatsApp. Indian Journal of Plastic Surery. 2013;46(3):502–505. 10.4103/0970-0358.121990 PMC3897093 24459338

[9] Giordano V, Koch HA, Mendes CH, Bergamin A, and de Souza FS, do Amaral NP. WhatsApp Messenger is useful and reproducible in the assessment of tibial plateau fractures: Inter- and intra-observer agreement study. Int. J. Med. Inform. 2015;84(2):141–148. 10.1016/j.ijmedinf.2014.11.002 25468642

[10] Astarcioglu MA, Sen T, Kilit C, Durmus HI, Gozubuyuk G, Kalcik M. et al Time-to reperfusion in STEMI undergoing inter hospital transfer using smartphone and WhatsApp messenger. Am. J. Emerg. Med. 2015;33(10):1382–1384. 10.1016/j.ajem.2015.07.029 26299691

[11] Giordano V, Koch H, Godoy-Santos A, Dias Belangero W, Esteves Santos Pires R, Labronici P. WhatsApp Messenger as an Adjunctive Tool for Telemedicine: An Overview. Interact Journal of Medical Research. 2017; 6(2):11. 10.2196/ijmr.6214 PMC5544893 28733273

[12] Lee RS, Woods R, Bullard M, Holroyd BR, Rowe BH. Consultations in the emergency department: a systematic review of the literature. Emergency Medicine Journal. 2008; 25(1): 4–9. 10.1136/emj.2007.051631 18156528

[13] Raiman L, Antbring R, Mahmood A. WhatsApp messenger as a tool to supplement medical education for medical students on clinical attachment. BMC Medical Education. 2017;17(1):7 10.1186/s12909-017-0855-x 28061777 PMC5219809

[14] Kaliyadan F, Ashique KT, Jagadeesan S, Krishna B. What’s up dermatology? A pilot survey of the use of WhatsApp in dermatology practice and case discussion among members of WhatsApp dermatology groups. Indian Journal of Dermatology, Venereology, and Leprology. 2016;82(1):67–9. 10.4103/0378-6323.171638 26728815

[15] Boulos MNK, Giustini DM and Wheeler S. Instagram and WhatsApp in Health and Healthcare: An Overview. Future Internet. 2016;8(37).

[16] Jagannathan M. Efficacy of communication amongst staff members at plastic and reconstructive surgery section using smartphone and mobile WhatsApp. Indian Journal of Plastic Surgery. 2013;46(3):506–507. 24459339 PMC3897094

[17] Khanna V, Sambandam SN, Gul A, Mounasamy V. et al "WhatsApp" ening in orthopedic care: a concise report from a 300-bedded tertiary care teaching center, European Journal of Orthopaedic Surgery & Traumatology. 2015;25(5):821–826. 25633127 10.1007/s00590-015-1600-y

[18] Choudhari P. Study on effectiveness of communication amongst members at department of orthopedics surgery unit 3 using smartphone and mobile WhatsApp, International Surgery Journal. 2014;1(1):9–12.

[19] Migliore M. The use of smartphones or tablets in surgery. What are the limits?, Annali Italiani di Chirurgia. 2015;86(2):185–186. 25953242

[20] Dhuvad JM, Dhuvad MM, Kshirsagar RA. Have Smartphones contributed in the clinical progress of oral and maxillofacial surgery? Journal of Clinical and Diagnostic Research. 2015;9(9):ZC22–ZC24. 10.7860/JCDR/2015/14466.6454 26501006 PMC4606335

[21] Pandian SS, Srinivasan P, Mohan S. The maxillofacial surgeon’s march towards a smarter future-smartphones. Journal of Maxillofacial and Oral Surgery. 2014;13:355–358. 10.1007/s12663-013-0497-4 26224996 PMC4518770

[22] Petruzzi M, De Benedittis M. WhatsApp: A telemedicine platform for facilitating remote oral medicine consultation and improving clinical examinations. Oral Surgery, Oral Medicine, Oral Pathology and Oral Radiology. 2016;121(3):248–254. 10.1016/j.oooo.2015.11.005 26868466

[23] Kelahmetoglu O, Firinciogullari R, Yagmur C. Efficient utility of Whatsapp: from computer screen to the surgeon’s hand to determine maxillofacial traumas. Journal of Craniofacial Surgery. 2015;26:1437. 10.1097/SCS.0000000000001627 26080228

[24] Suliman MT. Sending photos through WhatsApp: A faster method for teleconsultation, Journal of Local and Global Health Science. 2014; 2.

[25] Graziano F, Maugeri R, Iacopino DG. Telemedicine versus WhatsApp: from tradition to evolution,Neuroreport. 2015;26:602–603. 10.1097/WNR.0000000000000393 26053704

[26] Martyn-Hemphill C., Sarkar S., Withington J., Roy A., Cohen D., Green J.S.A. et al Whatsapp doc?' evaluating a novel modality of communication amongst urology team members to promote patient safety. The Journal of Urology. 2015;193:e169–e170.

[27] Watson L, Pathiraja F, Depala A, O'Brien B, Beyzade S. Ensuring safe communication in health care: a response to Johnston et al on their paper ‘‘Smartphones let surgeons know WhatsApp: an analysis of communication in emergency surgical teams”. Am J Surg. 2016;211(1):302–3. 10.1016/j.amjsurg.2015.04.017 26184352

[28] Davis F. Perceived usefulness, perceived ease of use, and user acceptance of information technology. MIS Quarterly. 1989;13(3):319–340.

[29] Moore G., & Benbasat I. Development of an instrument to measure the perceptions of adopting an information technology innovation. Information Systems Research. 1991;2(3):192–222.

[30] Scott WR. The Adolescence of Institutional Theory. Administrative Science Quarterly. 1987;32(4):493–511.

[31] Scott WR. Institutions and Organizations. 2nd ed Thousands Oaks (CA): Sage; 2001.

[32] Scott WR. Lords of the Dance: professionals as institutional agents. Organization Studies. 2008; 29:2(219–238).

[33] Agarwal R, Prasad J. Are Individual Differences Germane To The Acceptance Of New Information Technologies? Decision Science. 1999;30(2):361–391.

[34] Morris MG, Venkatesh V. Age Differences in Technology Adoption decision: Implications for a Changing Work Force. Personnel Psychology. 2000;53(2):375–403.

[35] O’Reilly MK, Nason GJ, Liddy S, Fitzgerald CW, Kelly ME, Shields C. DOCSS: doctors on-call smartphone study. Irish Journal of Medical Science. 2014;183(4):573–577. 10.1007/s11845-013-1053-4 24338079

[36] Venkatesh V & Davis FD. A Theoretical Extension of the Technology Acceptance Model: Four Longitudinal Field Studies. Management Science. 2000;46(2):186–204.

[37] Venkatesh V. Determinants of Perceived Ease of Use: Integrating Control, Intrinsic Motivation, and Emotion into the Technology Acceptance Model. Information Systems Research. 2000a;11(4):342–365.

[38] Venkatesh V, Morris MG, Davis GB, Davis FD. User Acceptance of Information Technology: Toward a Unifie d View. MIS Quarterly. 2003;27(3):425–478.

[39] Venkatesh V, Zhang X, Sykes TA. Doctors ‘Do Too Little Technology’ A Longitudinal Field Study of an Electronic Healthcare System Implementation. Information Systems Research. 2011;22(3):523–546.

[40] Scott WR. Institutionale Cerriers: Reviewing Models of Transporting Ideas over Time and Space and Considering Their Consequences. Industrial and Corporate Change. 2003;12(4):879–894.

[41] Pinzone M, Guerci M, Lettieri E, Redman T. Progressing in the change journey towards sustainability in healthcare: the role of ‘Green’ HRM. Journal of Cleaner Production. 2016;122:201–211.

