# Supplementary material for: WhatsApp in hospital? An empirical investigation of individual and organizational determinants to use
Source: PLoS One. 2019 Jan 11;14(1):e0209873. doi: 10.1371/journal.pone.0209873 (PMC6329505; doi:10.1371/journal.pone.0209873)
Supplement: S1 Table — (DOCX) [file pone.0209873.s001.docx]

**S1 Table. Questionnaire.**

| **Variables** | **Items/assumptions** |
| --- | --- |
| **Section 1: Use of WhatsApp** | |
| Use (personal) | I use WhatsApp to participate in group discussions |
|  | I use WhatsApp to send private messages to other people |
|  | I use WhatsApp to organize my agenda with others |
|  | I use WhatsApp to share moments of my life with others |
|  | I use WhatsApp to send written messages |
|  | I use WhatsApp to send images |
|  | I use WhatsApp to send audio notes |
|  | I use WhatsApp to send videos |
|  | I use WhatsApp even in front of other people |
|  | I connect to WhatsApp many times a day |
| Use (Colleagues) | I use WhatsApp to share scientific information with my colleagues |
|  | I use WhatsApp for manage and share the agenda with my colleagues |
|  | I use WhatsApp for the management of clinical trials |
|  | I use WhatsApp to communicate with my colleagues about clinical situations, without mentioning specific information of patients |
|  | I use WhatsApp to ask for information or give directions to my colleagues, without mentioning specific information of patients |
|  | I use WhatsApp to compare clinical data of specific patients with my colleagues, using patient data |
|  | I use WhatsApp to send patient data to my colleagues, in form of images or videos |
|  | I use WhatsApp to receive patient information from other hospitals |
| Use (with patients) | Some of my patients ask me to use WhatsApp to communicate with them |
|  | I suggest to some of my patients to use WhatsApp to communicate with me |
|  | I use WhatsApp to organize the agenda of appointments with my patients |
|  | I use WhatsApp to send to my patients the results of diagnostic tests |
|  | My patients send me pictures or videos via WhatsApp to get an evaluation before a visit |
|  | My patients send me via WhatsApp photos or videos to get an evaluation without having a scheduled visit |
|  | I use WhatsApp to prescribe drugs or treatments to my patients |
|  | I use WhatApp with chronic patients to monitor their clinical conditions |
|  | I use WhatsApp with patients to monitor the effects of certain drugs |
|  | I use WhatsApp to answer urgent questions that my patients ask me |
|  | I make clinical decisions based on information received via WhatsApp without further patient assessment |
| **Section 2: Scales and constructs of the proposed model** | |
| Individual variable: Perceived Usefulness | I am convinced that the use of WhatsApp improves communication |
|  | Using WhatsApp lets you know if the messages have been read by colleagues |
|  | To use WhatsApp for work is time saving because it is faster than phone or mail |
|  | I am convinced that if everyone used WhatsApp there would be a greater and more effective sharing of clinical knowledge |
|  | The use of WhatsApp can greatly contribute to reducing the costs in the Hospital |
|  | The use of WhatsApp has the limit of the need for internet connection |
|  | The use of WhatsApp at work reduces my productivity (eg: I am distracted by other factors that do not concern my job) |
|  | The use of WhatsApp positively affects my research activity (ie: it is easier to share data and results) |
|  | The use of WhatsApp positively affects my teaching activity |
|  | The use of WhatsApp for communication between health workers can increase the workload |
|  | The evaluation of images or videos sent via WhatsApp is not sufficient to make a diagnosis |
|  | Using WhatsApp to monitor patients' clinical conditions increases the likelihood of recovery of their clinical situation |
|  | Use of WhatsApp facilitates the doctor-patient relationship |
|  | Using WhatsApp in my work allows me to effectively exchange information with the patient, thus avoiding a medical examination |
| Regulative Factors | The hospital management asks me not to use WhatsApp among colleagues |
|  | The Hospital Management asks me not to use WhatsApp with patients |
|  | The Hospital Management asks me not to communicate sensitive patient data via WhatsApp |
| Normative Factors | My colleagues are using WhatsApp for personal reasons |
|  | My colleagues are using WhatsApp for professional reasons |
|  | My colleagues are using WhatsApp to share scientific information |
|  | My colleagues are using WhatsApp to communicate patient information |
|  | My colleagues do not want to use WhatsApp for professional reason |
|  | My patients ask me the use of WhatsApp |
|  | My patients prefer doctors who use WhatsApp |
|  | My patients are more likely to recover if they are using WhatsApp for care continuity |
| **Section 3: Control variables and characteristics of the respondent** | |
| General information | Age |
|  | Gender |
|  | Profession |
|  | Clinical Area or Unit |
|  | Academic role in this healthcare company |
|  | Work experience (indicate the number of years) |
|  | Work experience in this healthcare company (indicate the number of years) |
|  | What is the average age of your patients? |
|  | Do you have access to your smartphone at work? |
|  | What brand of smartphone do you use? |
|  | Use the Smartphone to communicate with patients? |
|  | How many years have you been using WhatsApp? |
|  | When do you use WhatsApp for work purposes? |
|  | How often do you use WhatsApp for work purposes? |
|  | When do you use WhatsApp for work purposes? |
|  | How often do you use WhatsApp for work purposes? |
|  | What is the percentage of the patients you follow who contact you via WhatsApp? |
|  | What is the average age of your patients using WhatsApp to communicate with her? |
| Perceived Risks | The use of WhatsApp to communicate patient data with other health professionals is safe and does not entail risks |
|  | Sending clinical data via WhatsApp involves risks for health professionals |
|  | The use of WhatsApp involves risks related to privacy and data protection |
|  | The use of WhatsApp carries the risk of uncontrolled spread of sensitive data |
|  | To communicate through WhatsApp involves clinical risks as it is not documented within the medical record |
|  | The use of WhatsApp for communication between patients and health professionals is safe and does not involve risks |
|  | The use of WhatsApp for communication can generate misunderstandings with the patient |
|  | Sending clinical-care data via WhatsApp involves risks for the patient |
|  | The use of WhatsApp involves the risk of incorrect clinical evaluations |
|  | The use of WhatsApp involves the risk of incorrect diagnosis and clinical decisions |
|  | The use of WhatsApp involves the risk of compromising the patient-physicians relationship |
|  | The use of WhatsApp for the transmission of sensitive data with the patient should provide consent for personal data treatment by the patient |
|  | The use of WhatsApp in the clinical setting is risky because no guidelines and recommendations are available about the safe mode of use and transmission of data |
