# Supplementary material for: WhatsApp in hospital? An empirical investigation of individual and organizational determinants to use
Source: PLoS One. 2019 Jan 11;14(1):e0209873. doi: 10.1371/journal.pone.0209873 (PMC6329505; doi:10.1371/journal.pone.0209873)
Supplement: S2 Table — (DOCX) [file pone.0209873.s002.docx]

**S2 Table. Personal use of WhatsApp.**

|  | | *Never* | *Rarely* | *Occasionally* | *Often* | *Always* | *p-value* |
| --- | --- | --- | --- | --- | --- | --- | --- |
| I use WhatsApp to participate in group discussions | Nurses | 13 | 16 | 35 | 50 | 11 | 0.68 |
|  | Physicians | 11 | 6 | 20 | 25 | 4 |  |
| I use WhatsApp to send private messages to other people | Nurses | 2 | 6 | 10 | 48 | 59 | 0.23 |
|  | Physicians | 3 | 3 | 7 | 32 | 21 |  |
| I use WhatsApp to organize my agenda with others | Nurses | 17 | 18 | 37 | 40 | 12 | 0.47 |
|  | Physicians | 13 | 14 | 17 | 17 | 4 |  |
| I use WhatsApp to share moments of my life with others | Nurses | 10 | 16 | 24 | 52 | 22 | 0.057 |
|  | Physicians | 11 | 16 | 10 | 23 | 6 |  |
| I use WhatsApp to send written messages | Nurses | 3 | 4 | 10 | 53 | 55 | 0.061 |
|  | Physicians | 6 | 1 | 6 | 35 | 18 |  |
| I use WhatsApp to send images | Nurses | 4 | 6 | 23 | 52 | 39 | 0.30 |
|  | Physicians | 5 | 5 | 14 | 29 | 13 |  |
| I use WhatsApp to send audio notes | Nurses | 13 | 18 | 34 | 32 | 27 | **< 0.0001** |
|  | Physicians | 18 | 18 | 19 | 8 | 3 |  |
| I use WhatsApp to send videos | Nurses | 11 | 21 | 32 | 31 | 29 | **0.003** |
|  | Physicians | 11 | 20 | 20 | 11 | 4 |  |
| I use WhatsApp even in front of other people | Nurses | 30 | 30 | 32 | 23 | 10 | 0.73 |
|  | Physicians | 15 | 17 | 17 | 15 | 2 |  |
| I connect to WhatsApp many times a day | Nurses | 7 | 19 | 35 | 42 | 20 | **0.05** |
|  | Physicians | 7 | 4 | 14 | 21 | 20 |  |
