# Supplementary material for: WhatsApp in hospital? An empirical investigation of individual and organizational determinants to use
Source: PLoS One. 2019 Jan 11;14(1):e0209873. doi: 10.1371/journal.pone.0209873 (PMC6329505; doi:10.1371/journal.pone.0209873)
Supplement: S3 Table — (DOCX) [file pone.0209873.s003.docx]

**S3 Table. Mode of WhatsApp usage between healthcare professionals.**

|  | | *Never* | *Rarely* | *Occasionally* | *Often* | *Always* | *p-value* |
| --- | --- | --- | --- | --- | --- | --- | --- |
| I use WhatsApp to share scientific information with my colleagues | Nurses | 22 | 36 | 41 | 22 | 4 | **0.038** |
|  | Physicians | 10 | 13 | 15 | 24 | 4 |  |
| I use WhatsApp for manage and share the agenda with my colleagues | Nurses | 10 | 18 | 46 | 39 | 10 | **0.001** |
|  | Physicians | 17 | 5 | 10 | 27 | 7 |  |
| I use WhatsApp for the management of clinical trials | Nurses | 55 | 34 | 22 | 10 | 2 | 0.097 |
|  | Physicians | 27 | 18 | 14 | 5 | 1 |  |
| I use WhatsApp to communicate with my colleagues about clinical situations, without mentioning specific information of patients | Nurses | 69 | 25 | 20 | 7 | 3 | **<0.0001** |
|  | Physicians | 17 | 16 | 15 | 14 | 4 |  |
| I use WhatsApp to ask for information or give directions to my colleagues, without mentioning specific information of patients | Nurses | 44 | 32 | 30 | 12 | 6 | **0.042** |
|  | Physicians | 17 | 15 | 13 | 18 | 3 |  |
| I use WhatsApp to compare clinical data of specific patients with my colleagues, using patient data | Nurses | 56 | 25 | 28 | 11 | 4 | 0.64 |
|  | Physicians | 33 | 14 | 9 | 8 | 2 |  |
| I use WhatsApp to send patient data to my colleagues, in form of images or videos | Nurses | 85 | 19 | 12 | 5 | 3 | **0.029** |
|  | Physicians | 30 | 17 | 13 | 4 | 2 |  |
| I use WhatsApp to receive patient information from other hospitals | Nurses | 105 | 7 | 7 | 3 | 2 | **0.001** |
|  | Physicians | 39 | 13 | 6 | 6 | 2 |  |
