# Supplementary material for: WhatsApp in hospital? An empirical investigation of individual and organizational determinants to use
Source: PLoS One. 2019 Jan 11;14(1):e0209873. doi: 10.1371/journal.pone.0209873 (PMC6329505; doi:10.1371/journal.pone.0209873)
Supplement: S4 Table — (DOCX) [file pone.0209873.s004.docx]

**S4 Table. Mode of WhatsApp usage with patients.**

|  | | *Never* | *Rarely* | *Occasionally* | *Often* | *Always* | *p-value* |
| --- | --- | --- | --- | --- | --- | --- | --- |
| Some of my patients ask me to use WhatsApp to communicate with them | Nurses | 102 | 14 | 4 | 1 | 0 | **<0.0001** |
|  | Physicians | 18 | 10 | 18 | 17 | 3 |  |
| I suggest to some of my patients to use WhatsApp to communicate with me | Nurses | 112 | 6 | 2 | 1 | 0 | **<0.0001** |
|  | Physicians | 41 | 10 | 6 | 6 | 3 |  |
| I use WhatsApp to organize the agenda of appointments with my patients | Nurses | 111 | 7 | 2 | 1 | 0 | **<0.0001** |
|  | Physicians | 39 | 11 | 9 | 6 | 1 |  |
| I use WhatsApp to send to my patients the results of diagnostic tests | Nurses | 117 | 2 | 2 | 0 | 0 | **0.001** |
|  | Physicians | 53 | 8 | 3 | 1 | 1 |  |
| My patients send me pictures or videos via WhatsApp to get an evaluation before a visit | Nurses | 114 | 3 | 2 | 2 | 0 | **<0.0001** |
|  | Physicians | 20 | 19 | 14 | 11 | 2 |  |
| My patients send me via WhatsApp photos or videos to get an evaluation without having a scheduled visit | Nurses | 114 | 3 | 2 | 2 | 0 | **<0.0001** |
|  | Physicians | 26 | 14 | 12 | 13 | 1 |  |
| I use WhatsApp to prescribe drugs or treatments to my patients | Nurses | 118 | 1 | 2 | 0 | 1 | **<0.0001** |
|  | Physicians | 52 | 11 | 1 | 2 | 0 |  |
| I use WhatsApp with chronic patients to monitor their clinical conditions | Nurses | 114 | 5 | 2 | 0 | 0 | **<0.0001** |
|  | Physicians | 42 | 11 | 9 | 4 | 0 |  |
| I use WhatsApp with patients to monitor the effects of certain drugs | Nurses | 117 | 2 | 2 | 0 | 0 | **<0.0001** |
|  | Physicians | 49 | 9 | 4 | 3 | 1 |  |
| I use WhatsApp to answer urgent questions that my patients ask me | Nurses | 111 | 3 | 6 | 1 | 0 | **<0.0001** |
|  | Physicians | 32 | 17 | 13 | 3 | 1 |  |
| I make clinical decisions based on information received via WhatsApp without further patient assessment | Nurses | 115 | 2 | 2 | 2 | 0 | **0.002** |
|  | Physicians | 54 | 8 | 3 | 0 | 1 |  |
