# Supplementary material for: WhatsApp in hospital? An empirical investigation of individual and organizational determinants to use
Source: PLoS One. 2019 Jan 11;14(1):e0209873. doi: 10.1371/journal.pone.0209873 (PMC6329505; doi:10.1371/journal.pone.0209873)
Supplement: S5 Table — (DOCX) [file pone.0209873.s005.docx]

**S5 Table. Perceived benefits about WhatsApp usage between healthcare professionals.**

|  | | *Totally disagree* | *Strongly disagree* | *Quite disagree* | *Neither agree nor disagree* | *Quite agree* | *Strongly agree* | *Totally agree* | *p-value* |
| --- | --- | --- | --- | --- | --- | --- | --- | --- | --- |
| I am convinced that the use of WhatsApp improves communication | Nurses | 6 | 8 | 20 | 16 | 49 | 17 | 7 | 0.73 |
|  | Physicians | 2 | 5 | 6 | 8 | 26 | 11 | 7 |  |
| Using WhatsApp lets you know if the messages have been read by colleagues | Nurses | 7 | 8 | 8 | 21 | 45 | 25 | 11 | 0.28 |
|  | Physicians | 1 | 3 | 1 | 15 | 20 | 21 | 5 |  |
| I am convinced that if everyone used WhatsApp there would be a greater and more effective sharing of clinical knowledge | Nurses | 17 | 18 | 15 | 30 | 22 | 12 | 6 | 0.97 |
|  | Physicians | 5 | 3 | 3 | 14 | 21 | 14 | 6 |  |
| The use of WhatsApp can greatly contribute to reducing the costs in the Hospital | Nurses | 17 | 13 | 20 | 41 | 16 | 9 | 3 | 0.17 |
|  | Physicians | 11 | 8 | 9 | 17 | 14 | 4 | 3 |  |
| The use of WhatsApp has the limit of the need for internet connection | Nurses | 15 | 9 | 11 | 32 | 26 | 17 | 9 | 0.29 |
|  | Physicians | 4 | 2 | 2 | 21 | 17 | 12 | 8 |  |
| The use of WhatsApp at work reduces my productivity (e.g.: I am distracted by other factors that do not concern my job) | Nurses | 9 | 12 | 17 | 24 | 29 | 24 | 8 | **0.046** |
|  | Physicians | 14 | 7 | 10 | 11 | 17 | 4 | 3 |  |
| The use of WhatsApp positively affects my research activity (e.g.: it is easier to share data and results) | Nurses | 17 | 12 | 16 | 30 | 23 | 16 | 5 | 0.26 |
|  | Physicians | 7 | 10 | 12 | 20 | 11 | 2 | 3 |  |
| The use of WhatsApp positively affects my teaching activity | Nurses | 11 | 13 | 13 | 41 | 18 | 17 | 5 | 0.053 |
|  | Physicians | 10 | 10 | 10 | 21 | 13 | 1 | 1 |  |
| The use of WhatsApp for communication between health workers can increase the workload | Nurses | 18 | 14 | 9 | 16 | 12 | 5 | 1 | >0.05 |
|  | Physicians | 12 | 9 | 7 | 20 | 11 | 4 | 3 |  |
