# Supplementary material for: WhatsApp in hospital? An empirical investigation of individual and organizational determinants to use
Source: PLoS One. 2019 Jan 11;14(1):e0209873. doi: 10.1371/journal.pone.0209873 (PMC6329505; doi:10.1371/journal.pone.0209873)
Supplement: S6 Table — (DOCX) [file pone.0209873.s006.docx]

**S6 Table. Perceived benefits related to WhatsApp usage with patients.**

|  | | *Totally disagree* | *Strongly disagree* | *Quite disagree* | *Neither agree nor disagree* | *Quite agree* | *Strongly agree* | *Totally agree* | *p-value* |
| --- | --- | --- | --- | --- | --- | --- | --- | --- | --- |
| The evaluation of images or videos sent via WhatsApp is not sufficient to make a diagnosis | Nurses | 10 | 11 | 8 | 34 | 20 | 22 | 9 | **0.008** |
|  | Physicians | 4 | 4 | 5 | 6 | 13 | 16 | 15 |  |
| Using WhatsApp to monitor patients' clinical conditions increases the likelihood of recovery of their clinical situation | Nurses | 10 | 16 | 5 | 19 | 32 | 17 | 20 | **0.045** |
|  | Physicians | 12 | 7 | 8 | 14 | 13 | 8 | 4 |  |
| Use of WhatsApp facilitates the doctor-patient relationship | Nurses | 19 | 7 | 13 | 29 | 21 | 18 | 8 | 0.53 |
|  | Physicians | 8 | 6 | 5 | 16 | 17 | 10 | 1 |  |
| Using WhatsApp in my work allows me to effectively exchange information with the patient, thus avoiding a medical examination | Nurses | 42 | 16 | 15 | 18 | 16 | 8 | 4 | 0.69 |
|  | Physicians | 21 | 11 | 11 | 11 | 10 | 2 | 0 |  |
