# Supplementary material for: WhatsApp in hospital? An empirical investigation of individual and organizational determinants to use
Source: PLoS One. 2019 Jan 11;14(1):e0209873. doi: 10.1371/journal.pone.0209873 (PMC6329505; doi:10.1371/journal.pone.0209873)
Supplement: S7 Table — (DOCX) [file pone.0209873.s007.docx]

**S7 Table. Perceived threats about WhatsApp usage between healthcare professionals.**

|  | | *Totally disagree* | *Strongly disagree* | *Quite disagree* | *Neither agree nor disagree* | *Quite agree* | *Strongly agree* | *Totally agree* | *p-value* |
| --- | --- | --- | --- | --- | --- | --- | --- | --- | --- |
| The use of WhatsApp to communicate patient data with other health professionals is safe and does not entail risks | Nurses | 33 | 25 | 28 | 9 | 22 | 4 | 2 | 0.41 |
|  | Physicians | 12 | 10 | 19 | 10 | 12 | 1 | 2 |  |
| Sending clinical data via WhatsApp involves risks for health professionals | Nurses | 7 | 2 | 8 | 14 | 45 | 27 | 15 | 0.17 |
|  | Physicians | 0 | 1 | 10 | 10 | 23 | 11 | 11 |  |
| The use of WhatsApp involves risks related to privacy and data protection | Nurses | 5 | 1 | 5 | 10 | 39 | 38 | 23 | 0.37 |
|  | Physicians | 0 | 3 | 4 | 6 | 22 | 16 | 14 |  |
| The use of WhatsApp carries the risk of uncontrolled spread of sensitive data | Nurses | 5 | 2 | 5 | 12 | 34 | 34 | 29 | 0.12 |
|  | Physicians | 1 | 5 | 2 | 9 | 25 | 11 | 11 |  |
| To communicate through WhatsApp involves clinical risks as it is not documented within the medical record | Nurses | 8 | 1 | 5 | 11 | 27 | 37 | 30 | **0.03** |
|  | Physicians | 1 | 5 | 1 | 8 | 20 | 11 | 18 |  |
