# Supplementary material for: WhatsApp in hospital? An empirical investigation of individual and organizational determinants to use
Source: PLoS One. 2019 Jan 11;14(1):e0209873. doi: 10.1371/journal.pone.0209873 (PMC6329505; doi:10.1371/journal.pone.0209873)
Supplement: S8 Table — (DOCX) [file pone.0209873.s008.docx]

**S8 Table. Perceived threats related to WhatsApp usage with patients.**

|  | | *Totally disagree* | *Strongly disagree* | *Quite disagree* | *Neither agree nor disagree* | *Quite agree* | *Strongly agree* | *Totally agree* | *p-value* |
| --- | --- | --- | --- | --- | --- | --- | --- | --- | --- |
| The use of WhatsApp for communication between patients and health professionals is safe and does not involve risks | Nurses | 39 | 25 | 31 | 11 | 10 | 4 | 2 | 0.86 |
|  | Physicians | 17 | 13 | 21 | 7 | 7 | 1 | 0 |  |
| The use of WhatsApp for communication can generate misunderstandings with the patient | Nurses | 6 | 0 | 10 | 11 | 47 | 33 | 13 | 0.21 |
|  | Physicians | 2 | 1 | 9 | 9 | 24 | 10 | 11 |  |
| Sending clinical data via WhatsApp involves risks for the patient | Nurses | 8 | 1 | 12 | 13 | 45 | 28 | 12 | **0.03** |
|  | Physicians | 0 | 2 | 12 | 9 | 26 | 7 | 10 |  |
| The use of WhatsApp involves the risk of incorrect clinical evaluations | Nurses | 5 | 0 | 8 | 14 | 41 | 33 | 20 | 0.20 |
|  | Physicians | 1 | 3 | 7 | 5 | 24 | 13 | 13 |  |
| The use of WhatsApp involves the risk of incorrect diagnosis and clinical decisions | Nurses | 6 | 1 | 11 | 15 | 39 | 32 | 17 | 0.29 |
|  | Physicians | 1 | 4 | 5 | 7 | 24 | 12 | 12 |  |
| The use of WhatsApp involves the risk of compromising the patient-physicians relationship | Nurses | 6 | 2 | 12 | 14 | 34 | 32 | 19 | **<0.0001** |
|  | Physicians | 3 | 12 | 9 | 14 | 13 | 7 | 8 |  |
| The use of WhatsApp for the transmission of sensitive data with the patient should provide consent for personal data treatment by the patient | Nurses | 6 | 2 | 3 | 16 | 25 | 34 | 36 | 0.27 |
|  | Physicians | 3 | 3 | 2 | 8 | 22 | 11 | 15 |  |
| The use of WhatsApp in the clinical setting is risky because no guidelines and recommendations are available about the safe mode of use and transmission of data | Nurses | 19 | 15 | 9 | 31 | 13 | 21 | 4 | **0.005** |
|  | Physicians | 5 | 3 | 3 | 17 | 11 | 13 | 13 |  |
